# Resistance mechanisms and fitness of pyraclostrobin-resistant isolates of *Lasiodiplodia theobromae* from mango orchards

**DOI:** 10.1371/journal.pone.0253659

**Published:** 2021-06-23

**Authors:** Rui He, Ye Yang, Zhizhong Hu, Ru Xue, Yajuan Hu

**Affiliations:** 1 Institute of Plant Protection, Hainan University, Haikou, China; 2 Hainan Medical University, Haikou, China; 3 Key Lab of Green Prevention and Control of Tropical Plant Diseases and Pests, Institute of Plant Protection, Hainan University, Haikou, China; Tocklai Tea Research Institute, INDIA

## Abstract

**Background:**

Stem-end rot, caused by *Lasiodiplodia theobromae* (Pat.) Griffon & Maubl is a serious postharvest disease in mango. In China, a high prevalence of the QoI fungicides resistance has been reported in the last decade. The study aimed to discuss factors determining rapid development of pyraclostrobin-resistance and its resistance mechanisms.

**Methods:**

To determine the resistance stability and fitness of pyraclostrobin resistance in *L*. *theobromae*, three phenotypes of pyraclostrobin resistance were compared and analyzed for the EC_50_ values, mycelial growth, virulence and temperature sensitivity and osmotic stress sensitivity. The relative conductivity and enzyme activities of different phenotypes were compared under fungicide stress to explore possible biochemical mechanisms of pyraclostrobin resistance in *L*. *theobromae*. The *Cytb* gene sequences of different phenotypes were analysed.

**Results:**

All isolates retained their original resistance phenotypes during the 10 subcultures on a fungicide-free PDA, factor of sensitivity change (FSC) was approximately equal to 1. The resistance-pyraclostrobin of the field isolates should be relatively stable. Two pyraclostrobin-resistant phenotypes shared similar mycelial growth, virulence and temperature sensitivity with pyraclostrobin-sensitive phenotype. After treated by pyraclostrobin, the relative conductivity of the sensitive phenotype was significantly increased. The time of Pyr-R and Pyr-HR reached the most conductivity was about 8–10 times than that of Pyr-S, the time for the maximum value appearance showed significant differences between sensitive and resistant phenotypes. The activities of Glutathione S-transferase (GST), catalase (CAT) and peroxidase (POD) of Pyr-HR were 1.78, 5.45 and 1.65 times respectively, significantly higher than that of Pyr-S after treated by 200 mg/l pyraclostrobin.

**Conclusion:**

The results showed that the pyraclostrobin-resistant phenotypes displayed high fitness and high-risk. The nucleotide sequences were identical among all pyraclostrobin-resistant and -sensitive isolates. The pyraclostrobin resistance was not attributable to *Cytb* gene alterations, there may be some of other resistance mechanisms. Differential response of enzyme activity and cell membrane permeability were observed in resistant- and sensitive-isolates suggesting a mechanism of metabolic resistance.

## Introduction

Mango (*Mangifera indica* L.) is one of the most important fruit crops in the tropical and subtropical regions. China is the second largest mango acreage in the world. In 2019, mango planting area in Hainan has exceeded 56,900 hectares and 675,805 tons. Stem-end rot (SER) of mango is a serious postharvest disease of mango [1–3]. Fruit infections start in the field at weak spots around the fruit stalk attachment where moisture accumulates and persists at first. After harvest, SER begins to develop with the advancement of fruit ripening, and may result in significant fruit decay and yield loss, the incidences of fruit disease are 10%-40% [4]. In Hainan province, SER is mostly caused by *Lasiodiplodia theobromae* Pat. (= *Botryodiploda theobromae* Pat.) [5, 6]. Furthermore, *L*. *theobromae* can cause more than 500 other plant diseases [7]. However, due to the lack of a resistant variety, chemical control is the most reliable prevention method available to mango farmers in China. Fungicide applications are the primary method of postharvest disease control in mango. Traditionally, synthetic fungicides are frequently used to control diseases (SER, anthracnose, powdery mildew, etc.) in the preharvest and postharvest periods. The field isolates of *L*. *theobromae* from mango have become resistant to benzimidazole fungicide carbendazim due to the long-term and extensive application [8–10]. Carbendazim-resistant isolates have become widespread in Hainan Province. Currently, both the quinone outside inhibitor (QoI) and sterol demethylation inhibitor (DMI) fungicides are used to control diseases of mango. To delay or avoid the development of fungicide resistance, the most widely adopted anti-resistance strategies are using mixtures of fungicides with different modes of action. QoI fungicides (such as azoxystrobin and pyraclostrobin) are the most important class of agricultural fungicides. Pyraclostrobin is among the newer members of QoI, possesses an extremely broad spectrum of activity. The mechanism of action is inhibition of electron transport between cytochrome b and cytochrome c1 in the mitochondrial respiratory chain, leading to disruption in the production of ATP [11]. Due to its single mode of action, QoI fungicides have a high risk of selection of resistance in phytopathogenic fungi, and field resistance to QoI fungicides has been reported in more than 30 species [12, 13]. There is known resistance to pyraclostrobin in various fungal species and also cross-resistance to other QoI fungicides. The Fungicide Resistance Action Committee (FRAC) has designated the QoI fungicides as being at a high risk of resistance development [14]. Fungicide resistance development is dependent mainly on mutations in the field and the inheritance of the new traits [15, 16]. Many studies indicated that the molecular mechanisms of resistance to QoI fungicide in various fungi are correlated with point mutations of *Cytb* gene [17, 18]. Furthermore, resistance stability, fitness, and competitive ability of field-resistant isolates are extremely important factors regarding the risk for the development of resistance [19, 20]. Many studies showed that a few of field isolates, particularly laboratory mutants are associated with fungicide resistance may concomitantly exert fitness costs. These resistant isolates suffered significant fitness penalties in mycelial growth, conidial germination, sclerotia production, virulence, or temperature sensitivity, and were less competitive than the sensitive isolates [21, 22].

Our previous studies indicated that the *L*. *theobromae* isolates of mango in Hainan province of China had high levels of resistance to QoI fungicides pyraclostrobin [9]. However, the resistance fitness costs, resistance mechanisms of *L*. *theobromae* to QoI remain unknown. Therefore, the objectives of this study were to compare the characteristics between pyraclostrobin-resistant and -sensitive isolates of *L*. *theobromae* to evaluate the resistance fitness and to reveal the resistance characteristics and mechanisms. The studies were to: (i) determine the fitness of pyraclostrobin-sensitive and -resistant *L*. *theobromae* isolates in order to assess the resistance risk by comparing the mycelial growth, virulence, temperature sensitivity and osmotic sensitivity; (ii) determine the role of biochemical mechanisms of pyraclostrobin resistance by comparing the cell membrane permeability, protein content and enzyme activities in isolates; (iii) analyse the *Cytb* gene sequence of pyraclostrobin-sensitive and -resistant *L*. *theobromae* isolates.

## Materials and methods

### Isolates of *Lasiodiplodia theobromae*

In our earlier lab study, *Lasiodiplodia theobromae* isolates were collected during a monitoring program to determine the sensitivity to the QoI fungicides. All isolates were isolated from diseased mango fruits in Hainan Province of China in 2014 and 2016, and 59% isolates were pyraclostrobin resistance. Details on the isolation procedure and the determination of sensitivity to fungicides have previously been described [9]. To study the fitness and biochemical properties of these isolates, 30 isolates were selected to test in the current study, including 5 sensitive isolates (Pyr-S, EC_50_ < 10 mg/l), 9 resistant isolates (Pyr-R, 10 mg/l ≤ EC_50_ < 100 mg/l) and 16 highly resistant isolates (Pyr-HR, EC_50_ ≥ 100 mg/l). The isolates were stored on potato dextrose agar (PDA) slants at 4°C until use.

### Determination of resistance stability

Pyraclostrobin technical (96% a.i., Hainan Zhengye Zhongnong High Technology Co., Ltd., Haikou, China) was dissolved in acetone to obtain 2.5×10^4^mg/l solutions and stored at 4°C. Salycyl hydroxamic acid (SHAM, 99% a.i., Shanghai Macklin Biochemical Co., Ltd., Shanghai, China) was dissolved in acetone to obtain 1×10^4^ mg/l solutions and stored at 4°C.

A 5-mm-diameter mycelia disc was excised from the edge of a 3-days-old colony and placed on a PDA plate without pyraclostrobin. After subcultured for 10 times, the EC_50_ values of 30 isolates to pyraclostrobin were calculated by probit analysis. The final concentration of pyraclostrobinin the PDA were adjusted to 0.69, 2.06, 6.17, 18.52, 55.56, 166.67 and 500 mg/l, and SHAM (the alternative oxidase inhibitor) was added to PDA at 100 mg/ml including controls without fungicide. The inhibition rates were converted to the probability values, and pyraclostrobin concentrations were log-transformed before use in a regression model. All isolates were incubated at 28°C for 36 h. Each isolate was cultured on three replicate plates per treatment. The experiment was performed independently three times. The stability of resistance was represented by the FSC value (factor of sensitivity change): FSC: the EC_50_ values of the 10th transfer divided by the EC_50_ values of the 1st transfer [15].

### Mycelial growth assay

A mycelia disc (5-mm diameter) was taken from the edge of a 3-day-old colony and placed on PDA plate, with three replicates per isolate. After incubation for 36 h in the dark at 28°C, the radial growth of the mycelial colonies was measured for each plate. The experiment was performed independently three times.

### Virulence assay

The virulence of each isolate with different pyraclostrobin-resistance phenotypes were evaluated on mango fruits. Fresh green unripe mango fruits of similar size (cultivar Guifei) were used for the evaluation purposes. The fruit surface was disinfested in 1% v/v sodium hypochlorite (NaOCl) solution for 1 min, rinsed three times with sterile distilled water, and air dried. Then, each fruit was lightly wounded at three places with a sterile needle, and mycelia discs (5-mm diameter) taken from 3-day-old PDA plates were placed on the wound sites. All fruits were incubated at 30 ± 2°C and 70 to 90% relative humidity. Five fruits per isolate were used, incubated with three mycelia discs per fruit. The lesion diameter was recorded every day. Based on the lesion diameter after incubated for 5 days, the field isolates were subsequently classified as: highly virulent isolates (lesions ≥ 30 mm), moderately virulent isolates (15 mm ≤ lesion < 30 mm) and weakly virulent isolates (lesions <15 mm).

### Temperature sensitivity assay

To determine the temperature sensitivity of *L*. *theobromae* with different pyraclostrobin-resistance phenotypes, a 5-mm mycelial plug was taken from the edge of a 3-day-old colony, transferred onto PDA plates and incubated separately at 5, 10, 15, 20, 25, 30, 35, or 40°C for 36 h in thermostatic incubators. The colony size was measured as previously described. Each isolate was cultured on three replicate plates per treatment. The experiment was performed independently twice.

### Osmotic sensitivity assay

To test the sensitivity to osmotic stress, a mycelial plug (5-mm diameter) was taken from the edge of a 3-day-old colony and transferred onto PDA plates containing 5, 10, 20, 40, 80, 100, or 150 g/l glucose, separately. In addition, the sodium chloride (NaCl) concentration in PDA was 0, 1.25, 2.5, 5, 10, 20, 40 or 80 g/l. Three plates of each treatment were incubated at 28°C for 36 h, and the colony size was measured as previously described. The experiment was performed independently twice.

### Test of cell membrane permeability

Cell membrane permeability of mycelia was represented by the relative conductivity [23]. For each isolate, mycelial plugs (5 mm diameter) from the margins of 3-day-old colonies on PDA were placed in 250 ml flasks (5 plugs per flask) containing 100 ml of PD broth and cultured at 120 rpm for 48 h. The mycelia of each isolate were collected on double gauze and rinsed twice with double-distilled water. After filtration in vacuum for 30 min, 1 g of mycelia per sample was suspended in 20 ml of double-distilled water containing pyraclostrobin at 100 mg/l. After 0, 5, 10, 20, 40, 80, or 120 min at 25°C, the electrical conductivity was measured with a conductivity meter to assess the extent of leaching of cell contents through cell membranes. After 120 min, the mycelia were boiled for 5 min, and final conductivity was measured. All tests had three replicates. The relative conductivity of mycelia was calculated as:

$$\text{Relative}\;\text{conductivity}\left(\%\right)=\text{Conductivity}/\text{Final}\;\text{conductivity}*100.$$

### Determination of protein content and enzyme activity

Preparation of tissue homogenate: Mycelia discs (5-mm diameter) from the margins of 3-day-old colonies on PDA were placed on new PDA plates containing 2, 20 or 200 mg/l pyraclostrobin. Control medium was not amended with fungicides. The mycelia grew substantially over the entire PDA plate after culturing at 28°C for 4 days. The mycelia were then scraped from the PDA and homogenized using nine volumes (1:9 w/v) of saline. Cell debris was removed by centrifuging at 4,000 rpm for 10 min, and the supernatant was used to assay the protein content and enzymatic activities.

Total protein content was determined using a Bradford protein assay kit (Nanjing Jiancheng Bioengineering Institute, Nanjing, Jiangsu, China). Total soluble protein was estimated by a dye-binding assay and monitored at 595 nm.

Enzyme activity was determined according to the kit instructions (Nanjing Institute of Bioengineering). The absorbance values of the respective tubes were measured by a spectrophotometer at room temperature, and the activity values of the relevant enzymes were calculated according to the formulae. Glutathione S transferase (GST) catalyzes the reduction of glutathione (GSH) to 1-chloro-2,4-dinitrobenzene. During the reaction time, the level of GST activity has a linear relationship with the change in GSH concentration before and after the reaction. The change in absorbance at 420 nm was measured to determine the amount of GSH. GST activity, as a measure of the reaction in which the concentration of GSH decreases by 1 μM per minute at 37°C, was expressed as units per milligram of total protein.

The reaction of catalase (CAT) to decompose hydrogen peroxide can be quickly stopped by adding ammonium molybdate. The remaining hydrogen peroxide reacts with ammonium molybdate to produce paleyellow complex, which is measured at 405 nm on a spectrophotometer. One unit of CAT was defined as the quantity of enzyme that liberated 1 mM hydrogen peroxide (H_2_O_2_) per minute per milligram of protein at 37°C.

Peroxidase (POD) activity detection method was based on the principle ofH_2_O_2_ reaction catalyzed by POD, and determined by an increase in absorbance at 420 nm with a spectrophotometer. One unit of POD was defined as the amount of enzyme that hydrolyzed 1 mg of substrate per minute per milligram of protein at 37°C.

Phenylalanine ammonia lyase (PAL) catalyzes the deamination reaction of phenylalanine, which releases NH_3_ to form trans-cinnamic acid. The change in absorbance at 290 nm caused by differential concentration of trans-cinnamic acid determines the activity of PAL. PAL activity of was expressed as units per mg of soluble protein.

### Nucleotide sequence analysis of the *Cytb* gene

The different pyraclostrobin-resistant phenotypes were used to analysis the *Cytb* gene. To extract the genomic DNA, each isolate was cultured on PDA at 28°C for 3 days. Mycelia were harvested and powdered under liquid nitrogen. The genomic DNA was extracted using MiniBEST plant genomic DNA extraction Kit (TaKaRa Bio inc., Dalian, China). Two primer pairs F 5’- TTATGGGTCATACAGAGC-3’ and R 5’-TACAATAGCAGGCGGAGT-3’ was adopted to amplify the *Ctyb* gene fragment containing the codon129, 137 and 143. All of the PCRs were performed in 20 μl volumes contained 10 μl of 2× Taq Master Mix, 1 μL of template DNA, 0.5 μL of each primer and 8 μL of ddH2O. The PCR conditions were 95°C for 3 min and then 35 cycles (95°C for 15 s, 55°C for 40 s, 72°C for 90 s), a final extension stage of 5 min at 72 °C. An approximately 500 bp single PCR fragment was amplified using this pair of primers. The PCR products were separated in 1.0% agarose gels in 1× TAE, and purified by using a DNA Purification Kit (Takara Bio Inc., Dalian, China). All PCR products were sequenced by Sangon Biotech (Shanghai, China) Co., Ltd. The sequences were subjected to using the BLAST and compared with those in the NCBI/ GenBank^®^ database. The sequences were annotated using BioEdit software (version 7.0.9) for manual editing and translation.

### Statistical analysis

Statistical analyses were performed with SPSS (SPSS Statistics 24.0, IBM, USA). Experimental data were checked for homogeneity of variances using Levene’s test, then the mean values ± standard deviations were calculated. In order to assess differences in mycelial growth, the relative conductivity or enzyme activity, the data were analyzed by one-way ANOVA of Completely Randomized design (CRD) followed by Tukey’s test (P < 0.05).

## Results

### Resistance stability

After 10 successive transfers on fungicide-free PDA medium, the EC_50_ values of Pyr-S, Pyr-R and Pyr-HR phenotypes ranged from 2.5 to 7.6 mg/l, 19.2 to 71.1 mg/l and 123.1 to 1375.2 mg/l, respectively. Thirty isolates retained their original resistance phenotypes during the 10 subcultures on a fungicide-free PDA. The change in EC_50_ for all isolates ranged from 0.7 to 1.4-fold (FSC ≈ 1, Table 1), indicating that the resistance-pyraclostrobin of *L*. *theobromae* field isolates was relatively stable.

**Table 1:** Resistance stability, mycelial growth and virulence in vitro for isolates of *Lasiodiplodia theobromae* differing in fungicide resistance.

Isolates	Phenotype^a^	EC_50_ values (mg/l)		FSC^b^	Mycelial growth^c^ (mm)	Lesion diameter^d^ (mm)	Virulence
		1st	10th				
CJJH20801	Pyr-HR	119.6	125.3	1.0	82.0±3.6ab	>40	high
CJTN10501	Pyr-HR	251.6	302.1	1.2	85.0±2.2a	32.4	high
CJTN30205	Pyr-HR	140.8	124.9	0.9	85.0±0a	>40	high
DTN83108	Pyr-HR	342.2	302.3	0.9	85.0±1.7a	38.6	high
DZTN10104	Pyr-HR	151.1	147.7	1.0	82.3±3.7b	17.1	moderate
JH21617	Pyr-HR	270.2	330.6	1.2	75.7±8.3bc	31.4	high
HLTN50801	Pyr-HR	106.8	110.3	1.0	85.0±0a	>40	high
HLTN51001	Pyr-HR	453.9	587.4	1.3	78.6±6.2bc	21.8	moderate
YCHJ80103	Pyr-HR	114.9	166.9	1.5	85.0±3.7a	>40	high
YCHJ80401	Pyr-HR	133.3	123.1	0.9	62.3±4.7d	10.6	low
YCHJ90103	Pyr-HR	125.4	119.1	0.9	77.3±7.4bc	>40	high
YCJH70402	Pyr-HR	641.0	573.2	0.9	78.3±3.5bc	>40	high
YZHJ90301	Pyr-HR	181.5	185.7	1.0	73.6±5.1c	9.3	low
YZHJ90402	Pyr-HR	1240.5	1375.2	1.1	74.3±2.7c	>40	high
YZTN10301	Pyr-HR	194.9	202.4	1.0	82.3±3.4ab	>40	high
YCXY70702	Pyr-HR	259.2	230.3	0.9	85.0±0a	20.7	moderate
Isolates	Phenotype^a^	EC_50_ values (mg/l)		FSC^b^	Mycelial growth^c^ (mm)	Lesion diameter (mm)	Virulence
		1st	10th				
CJJH20103	Pyr-R	88.5	71.1	0.8	85.0±0a	>40	high
CJJH20505	Pyr-R	96.4	70.9	0.7	80.3±0abc	10.2	low
CJTN20301	Pyr-R	18.0	15.2	0.8	85.0±0a	>40	high
CJXY30601	Pyr-R	33.5	45.7	1.4	80.3±3.4abc	>40	high
HLTN10402	Pyr-R	22.2	19.2	0.9	85.0±0a	>40	high
JSJH10101	Pyr-R	18.8	24.9	1.3	67.7±7.4cd	22.1	moderate
LDJH	Pyr-R	31.8	30.5	1.0	75.6±6.2c	32.5	high
YCTN70103	Pyr-R	88.1	69.1	0.8	85.0±0a	32.3	high
YCXY70704	Pyr-R	31.1	37.1	1.2	82.7±2.3ab	24.7	moderate
DFTN40801	Pyr-S	3.2	2.5	0.8	64.7±4.6cd	16.2	moderate
DFTN41002	Pyr-S	4.1	3.4	0.8	85.0±2.3a	>40	high
YCJH70107	Pyr-S	7.2	7.6	1.1	85.0±0a	8.8	low
YCXY70602	Pyr-S	9.3	7.2	0.8	78.6±3.5c	20.9	moderate
YZTN10503	Pyr-S	3.1	2.5	0.8	82.7±3.7ab	>40	high

^a^S = sensitive, R = resistant, and HR = high resistant.

^b^FSC (factor of sensitivity change): the EC_50_ values of the 10th transfer divided by the EC_50_ values of the 1st transfe.

^c^Values are means (± standard deviation) of each isolate for three repetitions. Values within the same column with the same lowercase letter are not significantly different at the 0.05 level according to the Tukey’s test.

^d^The lesion diameter was measured after incubated for 5 days.

### Mycelial growth and virulence

Thirty isolates varied in mycelial growth and virulence to mango (Table 1), there were significantly difference among isolates (F = 7.964, *P* = 0.000 < 0.05). However, when compared as phenotype groups, the mycelial growth had no significant differences in the means of mycelial growth between resistant and sensitive phenotypes (F = 0.104, *P* = 0.903), with the average mycelial growth of Pyr-HR, Pyr-R and Pyr-S being 79.8, 80.7 and 79.2 mm in diameter, respectively.

**Fig 1:**
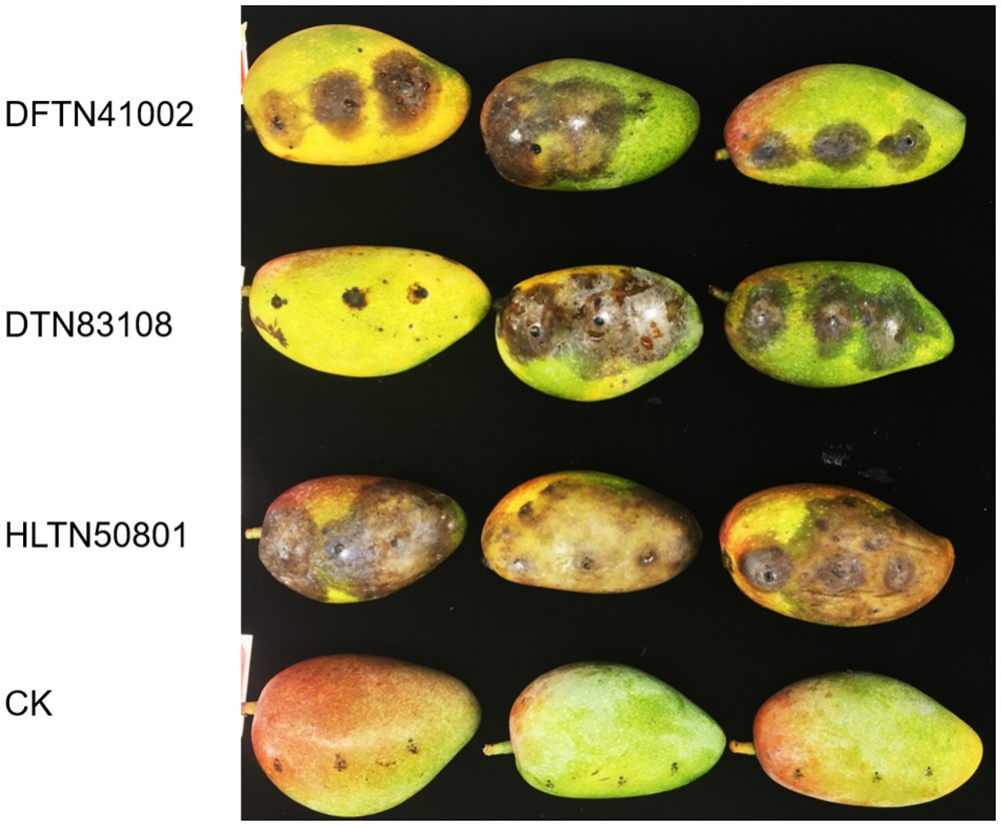
The virulence of *Lasiodiplodia theobromae* isolates at 5 d after inoculation.

When inoculated with *L*. *theobromae*, the brown to black lesions quickly developed on the mango fruits, and causing a watery rot, but control fruits remained healthy (Fig 1). The frequencies of highly virulent, moderately virulent and weakly virulent isolates were 63.3%, 23.3% and 13.3%, respectively. The virulence of pyraclostrobin-resistant (Pyr-HR and Pyr-R) isolates compared with that of pyraclostrobin-sensitive (Pyr-S) isolates did not significantly reduce.

### Sensitivity to temperature

All *L*.*theobromae* isolates grew faster after incubation at 25°C to 35°C. No isolate could grow on PDA at 5°C. When cultured at 10 °C, only 8 isolates grew after 48 h. The mycelial growth was significantly inhibited at 40°C. There was significant difference in mycelial growth between low- and high-temperature within the same phenotype groups (Table 2). When they were compared as phenotype groups, there was no significant difference between resistant and sensitive phenotypes in mean values of mycelial growth at 15 to 40°C after 36 h (*P* > 0.05).

**Table 2:** Sensitivity of *Lasiodiplodia theobromae* to different pyraclostrobin resistance phenotypes under different temperature (36h).

Phenotype	Growth (mm) at each Temperautre						Tukey’s test	
	15°C	20°C	25°C	30°C	35°C	40°C	F	*P*
Pyr-HR	17.6±7.4a	50.8±6.2b	81.2±3.78c	82.8±3.4c	79.3±6.2c	6.7±3.7a	121.43	0.000
Pyr-R	19.2±3.6b	50.7±3.3c	80.0±5.0d	83.9±1.7d	80.5±7.1d	5.6±3.5a	183.60	0.000
Pyr-S	18.3±6.3a	47.4±9.3b	80.8±8.3c	83.2±2.3c	79.7±8.0c	5.9±3.7a	75.78	0.000

Values are means (±SD) of three experiments. Values within the same row with the same lowercase letter are not significantly different at the 0.05 level according to the Tukey’s test. S = sensitive, R = resistant, and HR = high resistant.

### Sensitivity to osmotic stress

**Fig 2:**
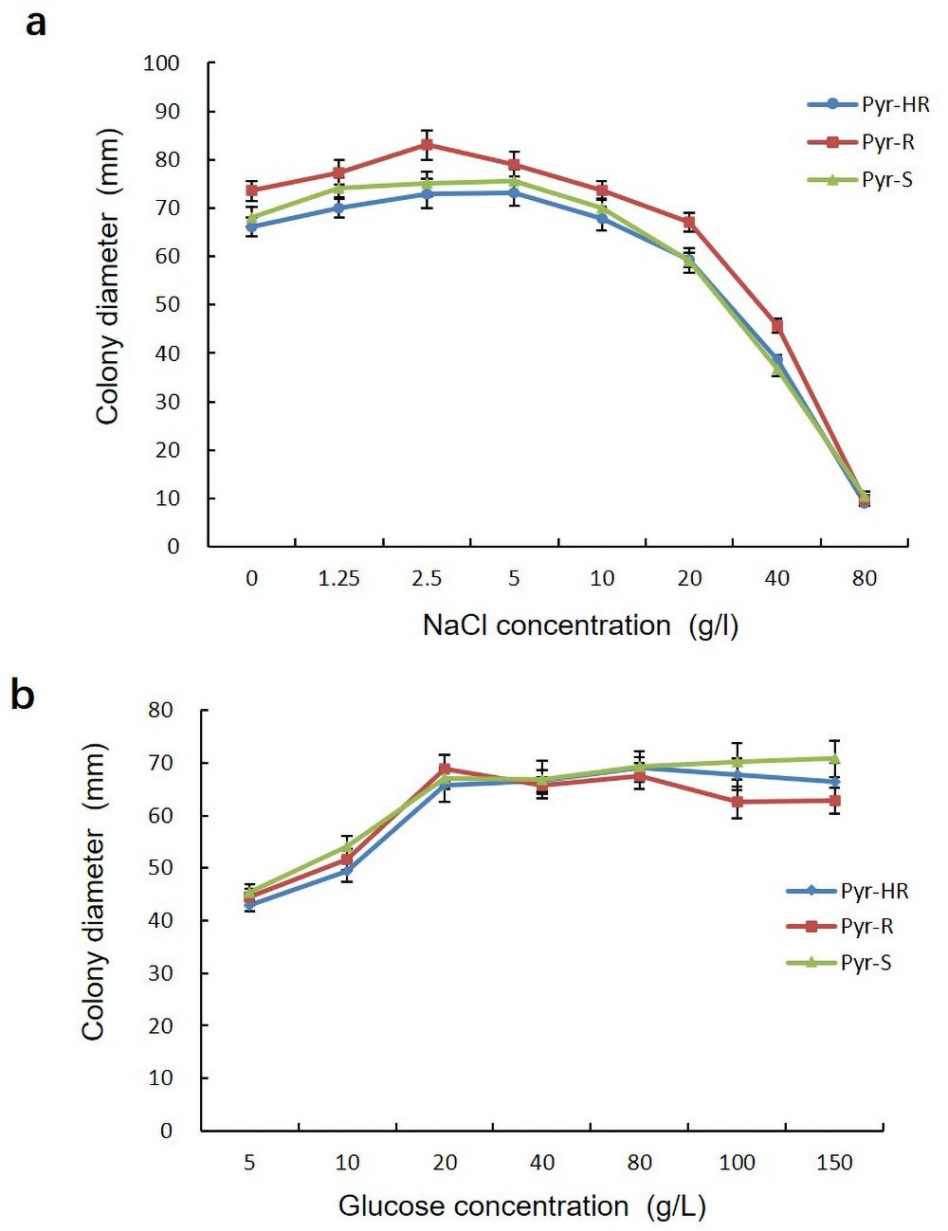
Osmotic stress sensitivity of *Lasiodiplodia theobromae* to different pyraclostrobin resistance phenotypes. (a) Sensitivity to NaCl. (b) Sensitivity to glucose. Bars denote the stand error of three experiments. S = sensitive, R = resistant, and HR = highly resistant.

The mycelial growth of different pyraclostrobin-resistant phenotypes increased slightly with increasing NaCl concentration from 0 to 2.5 g/l. However, the mycelial growth was significantly inhibited when the concentration increased above 5g/l (Fig 2a). For glucose at less than 20 g/l, the mycelial growth of all phenotypes increased significantly with increasing glucose concentration. There was little influence on mycelial growth when the glucose concentration increased above 20 g/l (Fig 2b). There were no significant differences among different phenotypes when growing in PDA with 80 g/l NaCl or 150 g/l glucose (F = 0.472, *P* = 0.645; F = 78.505, *P* = 1.000). After *L*. *theobromae* produced resistance to pyraclostrobin, the mycelial growth of Pyr-HR and Pyr-R phenotypes did not significantly decrease compared with Pyr-S phenotypes under osmotic stress.

### The relative conductivity and cell membrane permeability

**Fig 3:**
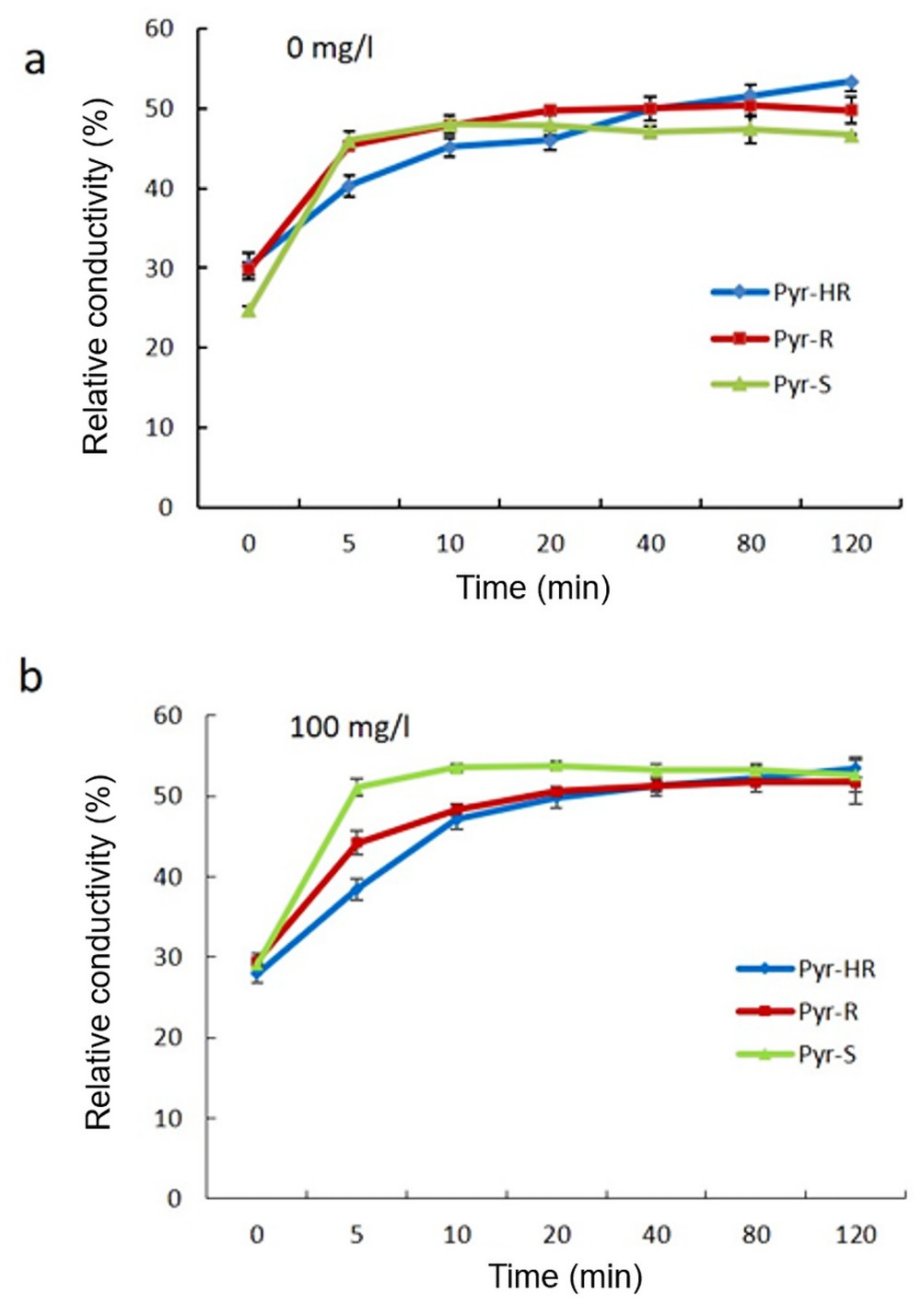
The relative conductivity of *Lasiodiplodia theobromae* to different pyraclostrobin resistance phenotypes. (a) Treated with no fungicede. (b) Treated with 100 mg/l pyraclostrobin. Bars denote the stand error of three experiments. S = sensitive, R = resistant, and HR = highly resistant.

The results showed that the relative conductivity of all *L*. *theobromae* was increased with time, the relative conductivity of three phenotypes reached the maximum after 80 min of treatment (Fig 3a). When treated by 100 mg/l pyraclostrobin, the relative conductivity of sensitive phenotype markedly increased in a short time (10 min), but resistant phenotypes required a longer time (≥ 80min) (Fig 3b). The relative conductivity of Pyr-S was significantly higher than that of Pyr-HR and Pyr-R after treated for 10 min (F = 50.09, *P* = 0.000 < 0.05). Cell membrane permeability was measured by the electrical conductivity. This means that cell membrane permeability of Pyr-S was markedly increased compared with that of the resistant phenotypes after treated by pyraclostrobin.

### Protein content and enzyme activity

The protein content of all phenotypes showed little variation with increasing pyraclostrobin concentrations. There were no significant differences between the resistant and sensitive phenotypes (*P* > 0.05).

**Fig 4:**
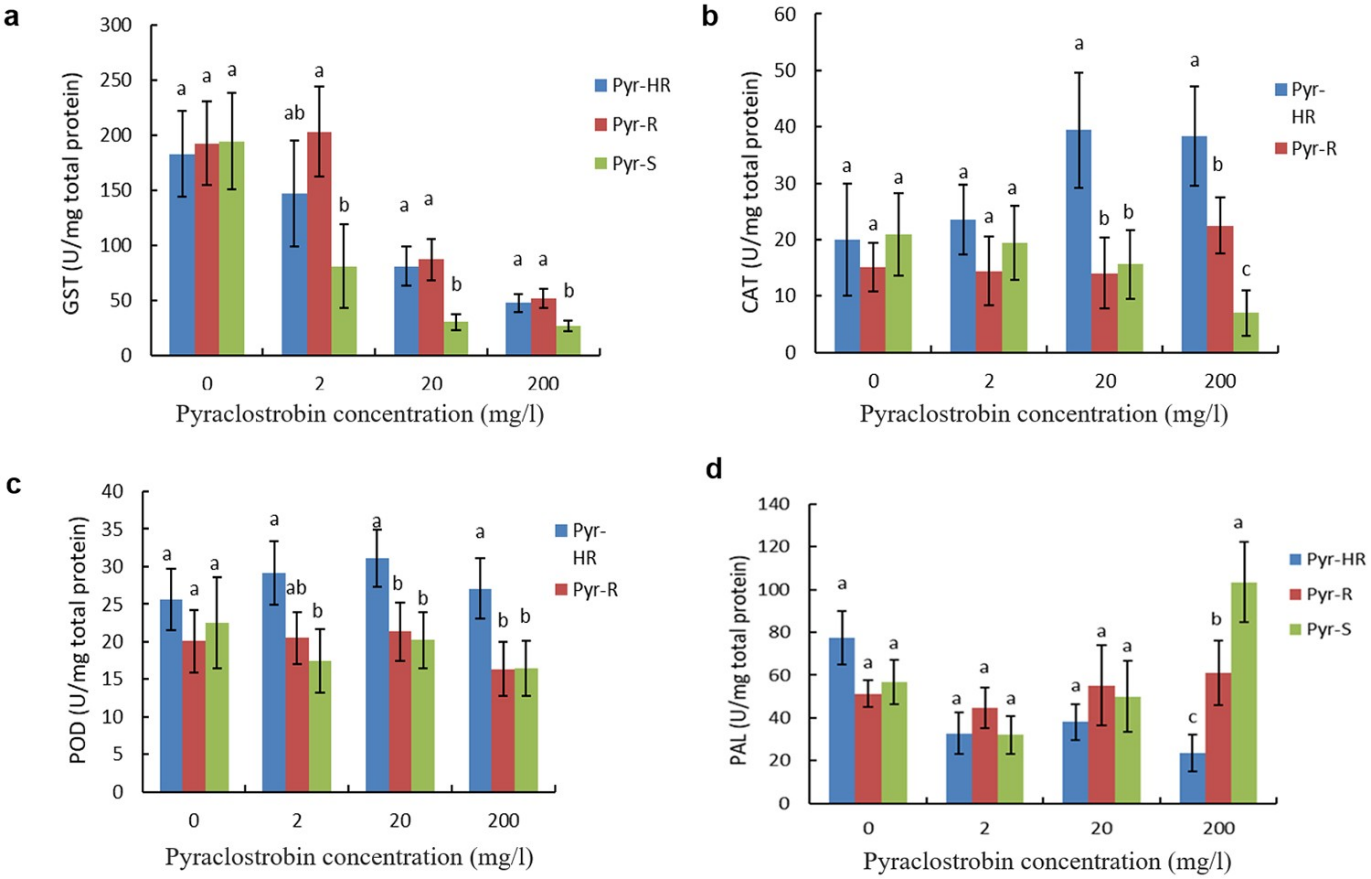
The enzyme activities in the mycelia of *Lasiodiplodia theobromae* phenotypes under fungicide stress. (a-d) The activities of GST, CAT, POD and PAL respectively. Bars denote the stand error of three experiments. S = sensitive, R = resistant, and HR = highly resistant.

The enzyme activities of *L*. *theobromae* isolates were measured after incubation with different concentrations of pyraclostrobin. GST activities of all phenotypes appeared notably decreased with an increase in fungicide treatment concentration. GST activities of the resistant phenotypes (Pyr-HR and Pyr-R) were significantly higher than that of the Pyr-S phenotype after treatment by pyraclostrobin (Fig 4a). Compared with that of control group, the decreasing rates of GST activity of Pyr-HR, Pyr-R and Pyr-S phenotypes were, respectively, 79.92%, 75.69% and 84.17% after treated by 200 mg/l pyraclostrobin. The GST activities of Pyr-HR and Pyr-R phenotypes were significantly higher compared with Pyr-S phenotype after treatment by 200 mg/l pyraclostrobin (F = 9.265, *P* = 0.015). But for the CAT activities, the Pyr-HR phenotype significantly increased with an increase in fungicide treatment concentration, and the Pyr-S phenotype displayed opposite result to Pyr-HR. (Fig 4b). The CAT activity of Pyr-HR phenotype was significantly higher compared with Pyr-S and Pyr-R phenotypes after treated by 200 mg/l pyraclostrobin (F = 18.623, *P* = 0.003). Similarly, the POD activities of Pyr-HR phenotype significantly increased with an increase in fungicide treatment concentration (Fig 4c). The POD activitie of Pyr-HR phenotype was significantly higher compared with Pyr-S and Pyr-R phenotypes after treatment by pyraclostrobin (F = 7.975, *P* = 0.02). For PAL activities, only Pyr-HR phenotype also appeared decreased with an increase in fungicide treatment concentration (Fig 4d). The PAL activities of Pyr-S phenotype were significantly higher compared with Pyr-R and Pyr-HR phenotypes after treated by 200 mg/l pyraclostrobin (F = 22.69, *p* = 0.002).

In summary, GST, CAT and POD activities of Pyr-HR phenotype of *L*. *theobromae* were significantly higher compared with Pyr-S phenotype in the high concentration pyraclostrobin that are contrary to PAL activity. The activities of GST, CAT and POD of Pyr-HR were 1.78, 5.45 and 1.65 times than that of Pyr-S, respectively.

### *Cytb* gene sequence

**Fig 5:**
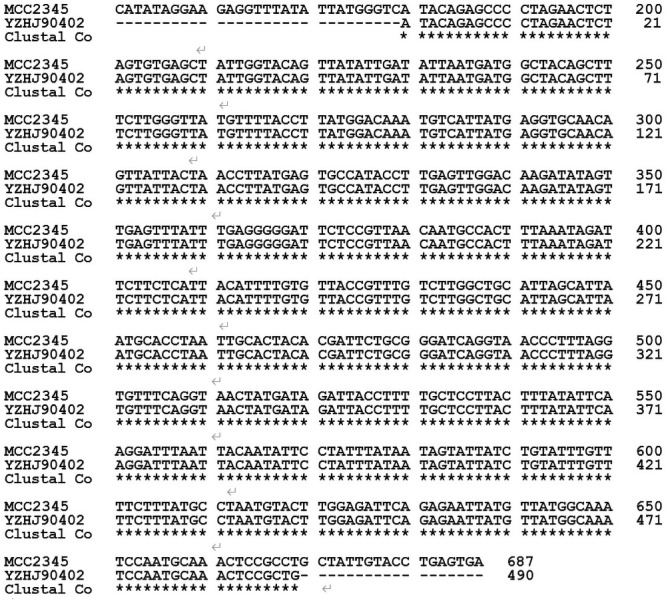
An alignment of the *Cytb* gene sequences compared with similar gene from other fungi. MCC2345: *Lasiodiplodia theobromae* isolate from papaya (GenBank: MH880818.1). YZHJ90402: pyraclostrobin resistance isolate of L. theobromae from mango.

Partial fragments were obtained from *Cytb* gene of *L*. *theobromae* isolates with 490 bp in length (Fig 5). BLAST search of the nucleotide sequence in GenBank showed 100% identity with *Cytb* gene in *L*. *theobromae* MCC2345 (GenBank: MH880818.1). The orthologous protein positions were aa 62 to 224. The sequenced fragment of the *Cytb* gene of *L*. *theobromae* includes the mutation positions known to affect the resistance of the pathogens to QoIs fungicides. There was no mutation observed in pyraclostrobin-resistant isolates. The results showed that all *L*. *theobromae* isolates had identical partial sequences, resistance to pyraclostrobin was not attributable to *Cytb* gene alterations.

## Discussion

It was reported that the wide occurrence of carbendazim and pyrazoxystrobin resistant isolates of *L*. *theobromae* in the Hainan mango growing region [4, 8, 9]. Resistance to site-specific fungicides has become a limitation to the sustained control of stem-end rot caused by *L*. *theobromae*. Knowledge of fungicide resistance is important in securing sustainable disease management in agricultural systems.

According to our previous study, QoI fungicide resistance can quickly develop in *L*. *theobromae* populations. In this study, the stability and fitness of pyrazoxystrobin-resistant isolates were analyzed to evaluate the risk of fungicide resistance. The results demonstrated that the pyraclostrobin-resistant isolates retained the same levels of EC_50_ values of pyraclostrobin as their initial generations after successive subculturing on PDA. It showed that pyraclostrobin resistance of *L*. *theobromae* was stable in the absence of the fungicide. However, there could be a difference between fields-resistant isolates and laboratory UV-induced mutants, the laboratory-induced mutants of *B*. *cinerea* almost completely lost pyraclostrobin resistance after 7 transfers on fungicide-free PDA [21]. Previous reports showed that some fungicide-resistant pathogen have sufficient fitness to compete with sensitive pathogen in the field [19, 20]. By contrary, some fungicide-resistant isolates may exert fitness costs, such as the carbendazim-resistant isolates were sensitive under low or high temperature conditions [22], the fludioxonil-resistant mutants were sensitive to osmotic stress [24], the pyraclostrobin-resistant mutants were less virulent than their sensitive wild parents [25]. In our study, the obvious difference of fitness was observed among isolates within the same phenotype groups, but there were no significant differences in the means among different phenotype groups. Two pyraclostrobin-resistant phenotypes shared similar mycelial growth, virulence and temperature sensitivity with pyraclostrobin-sensitive phenotype. And there were no significant differences among different phenotypes when grown in the presence of high NaCl and glucose concentration. These results showed that *L*. *theobromae* field isolates have no obvious fitness costs associated with pyraclostrobin resistance. There is a quite high and potential resistance risk of *L*. *theobromae* to pyraclostrobin. A similar pattern of results was obtained in carbendazim resistant isolates of *L*. *theobromae* [10]. It is further proved that *L*. *theobromae* is a kind of fungus with “high risk” for fungicide resistance development. Reduced sensitivity of fungicide target-sites and enhanced metabolic detoxification are the two major mechanisms in resistance development, such as the target gene point mutations [15–18], ATP-binding cassette transporters (ABC) overexpression [26], or changes of the activity of metabolic enzymes [27]. Recent studies have shown that fungicides could lead to cell membrane damage and mycelium electrolyte leakage increase in *Sclerotinia sclerotiorum* [28], and the fungicede-resistant isolates of *S*. *sclerotiorum* had a significant increase in relative conductivity [23, 29]. According to our study, the time that corresponds the maximal relative conductivity significantly differed among three phenotype groups after treated by pyraclostrobin; the time of Pyr-R and Pyr-HR reached the most conductivity was about 8–10 times than Pyr-S. There was significant difference between sensitive and resistant phenotypes when treated for 10 min. In summary, the relative electrical conductivity had a strong correlation with cell membrane permeability, so pyraclostrobin could lead to increased leakage from mycelium of sensitive isolates in a short time, but resistant isolates required a longer time. Therefore, it is speculated that the altered cell membrane permeability of the resistant isolates may be linked to fungicides resistance.

Numerous studies have demonstrated enzyme activities may play a role in the resistance of pesticides [30]. An increase in the enzyme activities was observed in fungicide-resistant isolates, which enhanced metabolic detoxification [31, 32]. Recent studies have reported that GST is the major enzyme classes involved in cell detoxification processes, it may be responsible for the carbendazim resistance of *Fusarium graminearum* [27]. Moreover, POD activity was significantly higher in the dimethachlone-resistant compared to the sensitive isolates of *S*. *sclerotiorum* [29]. In our study, GST activities of the resistant phenotypes in the untreated control group were not significantly different from that of the sensitive isolates. GST activities in the three phenotypes appeared to decrease with increasing pyraclostrobin concentration, but such a decrease in GST activity was obviously less in the resistant- than the sensitive-phenotypes. However, CAT activity of resistant isolates increased with an increase in the fungicide treatment concentration. The results indicated that changing rules on several enzymes activities of the resistant *L*. *theobromae* were different after treated with pyraclostrobin. It is important to note, no matter enzymes activities are increased or decreased with the increasing of pyraclostrobin concentration, the end result is that GST, CAT and POD activities of the resistant phenotype groups (especially Pyr-HR) were significantly higher than that of Pyr-S. Thus, it can be reasonably speculated that enzyme activities might have been linked to the QoI fungicides resistance mechanism in *L*. *theobromae*.

The primary mechanism of fungicide-resistance in phytopathogenic fungi is target gene mutations. The QoI fungicides resistance generally associated with point mutations in *Cytb* gene at codons 129,137 and 143 in many phytopathogens [12, 13, 33]. Others have shown that *Cytb* gene mutation was not also detected in few pathogens, such as reduced sensitivity to azoxystrobin *L*. *theobromae* isolates [34], low and moderate resistant *Colletotrichum gloeosporioides* isolates [35], moderate and high resistant *Microdochium majus* isolates [36]. Though *L*. *theobromae* from mango has high level resistance to pyraclostrobin, but no point mutation was detected in pyraclostrobin-resistant isolates in this study. Our results indicated that the resistance mechanism to pyraclostrobin is not based on *Cytb* gene modifications, there may be some of other resistance mechanisms in *L*. *theobromae*.

The present study provided the important reference data for assessment of resistance risk and resistance mechanisms of *L*. *theobromae*. The high fitness of the pyraclostrobin-resistant *L*. *theobromae* populations present serious obstacles for management of QoI fungicides resistance. Field resistance to both benzimidazole and QoI fungicides are distributed widely in the mango growing regions of Hainan province. The present results highlight the need for strategies to reduce the risk. Furthermore, it is necessary to strengthen the research on the resistance mechanisms. The results indicated that pyraclostrobin resistance for *L*. *theobromae* was not attributable to *Cytb* gene alterations. This study also provides some evidence that *L*. *theobromae* can escape from fungicide inhibition not only by detoxification, but also by other resistance mechanisms. In conclusion, it would appear that the metabolic resistance may play key roles in fungicide resistance. However, metabolic resistance is poorly understood in the field of fungicides. Consequently, more metabolic resistance studies (e.g. key metabolic enzymes and related genes, ABC transporter genes) will be conducted to confirm the mechanism of QoI fungicides resistance in *L*. *theobromae* field isolates in future.
